# Metformin Reduces Desmoplasia in Pancreatic Cancer by Reprogramming Stellate Cells and Tumor-Associated Macrophages

**DOI:** 10.1371/journal.pone.0141392

**Published:** 2015-12-07

**Authors:** Joao Incio, Priya Suboj, Shan M. Chin, Trupti Vardam-Kaur, Hao Liu, Tai Hato, Suboj Babykutty, Ivy Chen, Vikram Deshpande, Rakesh K. Jain, Dai Fukumura

**Affiliations:** 1 Edwin L. Steele Laboratories, Department of Radiation Oncology, Massachusetts General Hospital Research Institute, Harvard Medical School, Boston, Massachusetts, United States of America; 2 Department of Internal Medicine, Hospital S. Joao, Porto, Portugal; 3 I3S, Institute for Innovation and Research in Heath, Metabolism, Nutrition and Endocrinology group, Biochemistry Department, Faculty of Medicine, Porto University, Porto, Portugal; 4 Department of Botany and Biotechnology, St. Xaviers College, Thumba, Trivandrum, Kerala, India; 5 Program of Biology and Biomedical Sciences, Harvard Medical School, Boston, Massachusetts, United States of America; 6 Department of Surgery, Keio University School of Medicine, Tokyo, Japan; 7 Department of Zoology, Mar Ivanios College, Nalanchira, Trivandrum, Kerala, India; 8 School of Engineering and Applied Sciences, Harvard University, Cambridge, Massachusetts, United States of America; 9 Department of Pathology, Massachusetts General Hospital, Harvard Medical School, Boston, Massachusetts, United States of America; The University of Texas MD Anderson Cancer Center, UNITED STATES

## Abstract

**Background:**

Pancreatic ductal adenocarcinoma (PDAC) is a highly desmoplastic tumor with a dismal prognosis for most patients. Fibrosis and inflammation are hallmarks of tumor desmoplasia. We have previously demonstrated that preventing the activation of pancreatic stellate cells (PSCs) and alleviating desmoplasia are beneficial strategies in treating PDAC. Metformin is a widely used glucose-lowering drug. It is also frequently prescribed to diabetic pancreatic cancer patients and has been shown to associate with a better outcome. However, the underlying mechanisms of this benefit remain unclear. Metformin has been found to modulate the activity of stellate cells in other disease settings. In this study, we examine the effect of metformin on PSC activity, fibrosis and inflammation in PDACs.

**Methods/Results:**

In overweight, diabetic PDAC patients and pre-clinical mouse models, treatment with metformin reduced levels of tumor extracellular matrix (ECM) components, in particular hyaluronan (HA). *In vitro*, we found that metformin reduced TGF-ß signaling and the production of HA and collagen-I in cultured PSCs. Furthermore, we found that metformin alleviates tumor inflammation by reducing the expression of inflammatory cytokines including IL-1β as well as infiltration and M2 polarization of tumor-associated macrophages (TAMs) *in vitro* and *in vivo*. These effects on macrophages *in vitro* appear to be associated with a modulation of the AMPK/STAT3 pathway by metformin. Finally, we found in our preclinical models that the alleviation of desmoplasia by metformin was associated with a reduction in ECM remodeling, epithelial-to-mesenchymal transition (EMT) and ultimately systemic metastasis.

**Conclusion:**

Metformin alleviates the fibro-inflammatory microenvironment in obese/diabetic individuals with pancreatic cancer by reprogramming PSCs and TAMs, which correlates with reduced disease progression. Metformin should be tested/explored as part of the treatment strategy in overweight diabetic PDAC patients.

## Introduction

The prognosis for patients with pancreatic cancer is dismal, with an overall five-year rate survival of 7% [1]. Obesity and type-2 diabetes mellitus (DM2) have become a pandemic worldwide [2, 3]. Recent studies have demonstrated that these metabolic abnormalities are associated with the increased incidence, progression and poor prognosis of PDAC [4–9]. At diagnosis, approximately half of PDAC patients are overweight or obese, and up to 80% of patients present with diabetes or glucose intolerance [10–17]. DM2 and obesity may promote PDAC through pro-tumorigenic insulin and insulin-like growth factor-1 (IGF-1) [18–20] as well as chronic inflammation [21, 22]. Hence, pharmacological interventions that target diabetes/obesity may also produce anti-tumor effects. One such agent, currently under intense investigation, is metformin, the most widely prescribed anti-diabetic generic drug that is also frequently administered to diabetic PDAC patients [23]. Metformin has been shown to improve treatment outcomes in preclinical models of PDAC [24–30]. In addition, it reduces the incidence of pancreatic cancer in diabetic patients as well as improves survival (reduced risk of death by 32%) in newly diagnosed cases [31–33]. However, the mechanisms of action of metformin in pancreatic cancer are not well understood. *In vitro* studies have addressed the impact of metformin on transcription factors, microRNAs, DNA damage, cancer stem cells and metabolism [34–38]. In addition, metformin has been shown to modulate the function of hepatic stellate cells, reduce oxidative stress in cancer-associated fibroblasts, and decrease tumor inflammation [34, 35, 39, 40]. We and others have shown that reprogramming PSCs reduces the production of extracellular matrix (ECM) components such as collagen-I and hyaluronan (HA), and slows the progression of pancreatic cancer [41–45]. However, the impact of metformin on PSC activation, production of ECM components and tumor desmoplasia has never been described.

The aim of this study is to elucidate the functional mechanisms of metformin within the PDAC fibro-inflammatory tumor microenvironment. *In vivo* studies of pancreatic cancers to date have been mainly performed in xenograft models in normal weight/non-diabetic mice where metformin has been shown to be less effective [26, 46–48]. Hence, we used two immunocompetent syngeneic mouse models that closely mimic obesity / DM2, to better represent pancreatic cancer patients—the majority of pancreatic cancer patients present with either new onset DM2 or impaired glucose tolerance at the time of diagnosis [10–17]—and the target population of metformin. Furthermore, we complemented our mouse models with *in vitro* studies as well as with analysis of human samples of pancreatic cancer from normal weight and overweight/obese patients. We found that metformin directly reprograms PSCs and TAMs and alleviates fibrosis and inflammation in obese/diabetic models of PDAC. These effects of metformin correlated with reduced ECM remodeling and epithelial-to-mesenchymal transition (EMT), ultimately affecting metastasis. We confirmed that ECM levels in human PDAC samples in overweight and obese patients were indeed lower in the patient population treated with metformin.

## Materials and Methods

### Animal experiments

The Institutional Animal Care and Use Committee of the Massachusetts General Hospital approved all experimental use of animals in this study. All animal procedures followed Public Health Service Policy on Humane Care of Laboratory Animals guidelines. Wild-type (WT) C57BL/6 and FVB male mice were originally obtained from The Jackson Laboratory (The Jackson Laboratory, Bar Harbor, Maine) and bred and maintained in our defined-flora animal facility. Mice were maintained on a 12-h light-dark cycle in a temperature-controlled barrier facility, with *ad libitum* access to food and acidified water. To generate obese/diabetic mouse models, mice (6-weeks old) were given a 60% fat diet (D12492, Research Diets, New Brunswick, NJ) for 10 weeks as previously described [49–51] (time of tumor implantation) and continued for the duration of tumor studies. For tumor experiments, the PAN02 and AK4.4 syngeneic PDAC models were used in C57BL/6 and FVB immunocompetent mice, respectively. PAN02 cells (*SMAD4-m174*) [52] were obtained from ATCC. AK4.4 cells (*KrasG12D* and *p53* ^*+/-*^) were kindly provided by Dr. Nabeel Bardeesy at MGH. These cells were isolated from mice generating spontaneous pancreatic tumors (*Ptf1-Cre/LSL-Kras* ^*G12D*^ */p53* ^*Lox/+*^) [53]. Orthotopic pancreatic tumors were generated by implanting a small piece (1 mm^3^) of viable tumor tissue (from a source tumor in a separate donor animal) into the pancreas of male lean or obese FVB (AK4.4 model) or C57BL/6 (PAN02 model) mouse. Both tumor models used were authenticated by IDEXX Laboratories. (PAN02: IDEXX RADIL Case # 22366–2013. AK4.4: IDEXX RADIL Case # 27818–2014).

### Pancreatic tumor growth studies

At 7 days post implantation, mice bearing orthotopic PAN02 or AK4.4 pancreatic tumors were randomized into control or metformin treatment groups. At day 21, plasma and tumor samples were collected, and tumors were weighed and processed for further analysis.

### Metformin treatment

The standard dose of metformin for treating humans is 1000 to 2500 mg, usually given twice daily. In the present study, metformin was administered at 300 mg/kg in drinking water. This can be translated to the human equivalent dose by using the Reagan-Shaw method [54]. According to the formula for the human equivalent dose [(mg/kg) = animal dose (mg/kg) x animal (km)/ human (km). Km for a 60 kg human adults equals 37 and for a 20 g mouse equals 3], the human equivalent of murine dose of 300 mg/kg is 1459 mg for an average sized 60 kg adult human. Therefore, the selected dose in the present study is within the safe therapeutic range recorded in humans. Furthermore, the present study determined the therapeutic period to be 2 weeks to evaluate the antitumor effect of metformin. This was compatible with the therapeutic periods reported in previous studies [55, 56]. Fresh metformin was administered in drinking water every 3 days. The amount added to each animal cage was calculated based on the average daily water intake for that cage during a period of 2 weeks prior to treatment initiation, and adjusted every three days based on water consumption and body weight of the animals. The approximate plasma concentration of metformin in patients with type 2 diabetes taking this drug is 0.05 mM, although it may accumulate in tissues and reach higher concentrations locally [35]. For *in vitro* experiments, a range of concentration from 0.05 to 25 mM depending on the cell line used (please see below for more details).

### Effect of metformin on PSCs and macrophages *in vitro*

Standard MTT assays were performed on PSCs and macrophages treated with metformin in a range of 0.05-25mM, to examine the potential effects on cell viability. RAW 264.7 (mouse leukemic monocyte-macrophages) were obtained from ATCC and used to assess the effect of metformin on macrophages *in vitro*. Cells were seeded in 10 cm^2^ petri dishes in serum/serum-free media and treated with metformin for 48h at concentrations ranging from 0.05 to 0.4 mM (concentrations that do not substantially affect cell viability). Following treatment, cells were collected for RNA and protein extraction in order to perform subsequent analysis of gene expression of cytokines and polarization markers, and for analysis of signaling and metabolic pathways. Human PSCs were seeded in 10 cm^2^ petri dishes in media with 2.5% serum and treated with metformin for 48h at concentrations ranging from 0.1 to 10 mM. Cells were collected for protein extraction for analysis of the activation of fibrosis-related pathways. Additionally, PSCs were seeded in an 8-well chamber slide (20,000 cells/well), treated with metformin (1 mM, a concentration that does not affect cell viability) for 48h, and immunofluorescent staining was performed following standard protocols. The cells were fixed with 4% paraformaldehyde and blocked with 5% normal donkey serum for 1 h. They were incubated with the designated primary antibodies overnight at 4°C then for 2h with the appropriate secondary antibodies at RT. PBS was used for all washes, and the stained samples were mounted with Prolong Gold with DAPI. Images were acquired using a confocal microscope. The antibodies used and image acquisition settings are described below. Both cell types were grown in Dulbecco's modified Eagle's medium (Sigma, St. Louis, MO) supplemented with 5% (v/v) heat-inactivated fetal bovine serum (FBS; Sigma), 100 units/ml penicillin and streptomycin. Cells were cultured at 37°C in a humidified atmosphere including 5% CO_2_.

### Human samples

Human samples of pancreatic cancer were obtained from the MGH tissue repository (http://www.massgeneral.org/research/resourcelab.aspx?id=31) under an active IRB protocol (Partners Healthcare IRB approval number: 2013P001969). Written informed consent from the donor or the next of kin was obtained for the use of these samples for research purposes. Tumors selected received no prior chemotherapy or radiation therapy before the surgical specimen was collected at the time of tumor resection. A total of 28 samples were randomly selected from this subset of samples. Body mass index was obtained for the respective sample. 7 of the samples (25%) correspond to patients that were medicated with metformin at the time of resection. Paraffin sections were stained for collagen-I and HA as described below. Images were acquired using confocal microscopy and quantified using Matlab. Data were analyzed anonymously.

### Gene Expression

Immediately following excision, tumor tissue was snap-frozen and stored in liquid nitrogen. Total RNA was extracted and relative gene expression of macrophage M1/M2 markers was determined using a Sybr-green based standard protocol. In addition, total RNA was extracted, and relative gene expression was determined using RT2 Profiler PCR Arrays system (Qiagen) on a Stratagene Mx3000P QPCR System using pre-made pathway-focused arrays (“Fibrosis”—Cat. Number: PAMM011Z; “Epithelial to Mesenchymal Transition (EMT)”—Cat. Number: PAMM090Z; “TGF-ß signaling targets”—PAMM235Z; “Extracellular Matrix & Adhesion Molecules”—Cat. Number: PAMM013Z; and “Common cytokines “—Cat. Number: PAMM021Z).

### Protein Expression

#### Western blot analysis

Each tumor sample was homogenized directly in lysis buffer for protein extraction. 20ug of denatured protein per sample was loaded on 7%, 10%, and 12% SDS-polyacrylamide gels. Antibodies used: phospho-p38 MAPKT^180/Y182^ and p38; phospho-JNK (SAPK/JNK)^Thr183/Tyr185^ and JNK; phospho-AKT^Ser473^ and AKT, phospho-ERK(p44/42 MAPK)^T202/Y204^ and ERK; phospho-NF-κB^Ser536^ and NK-κB; p65^Ser536^; phospho-Smad2^Ser465/467^ and Smad2; phospho-IGF-I Receptor ß^Tyr1135^ and IGF; Phospho-IRS-1^Ser612^ and IRS; IR; phpspho-stat3^Tyr705^ and stat3; Phospho-AMPKα^Thr172^ and Ampkα; phospho-Ampkβ^Ser108^ and Ampk; phospho-ACC^Ser79^ and ACC; Phospho-PDGF Receptor ß^Tyr751^ and PDGF Receptor ß and snail, e-cadherin and vimentin, TGF-ß, ß-catenin, twist, LC3B. All antibodies listed above were obtained from Cell Signaling Technology (Beverly, MA), and diluted 1:1000 with the exception of phospho-JNK (SAPK/JNK)^Thr183/Tyr185^, Phospho-NF-κB p65^Ser536^, phospho-Smad2^Ser465/467^, phospho-IGF-I Receptor ß ^Tyr1135^, Cleaved caspase-3, Phospho-IRS-1^Ser612^. Other antibodies used were: αSMA and phospho-Insulin Receptor^Y972^ (1:1000 and 1:500, Abcam, MA); col-1 (1:1000); MMP-9 and MMP-2 (1:500 and 1:200, EMD Millipore-Billerica, MA), AT1 (1:1000, LifeSpan BioSciences Inc, WA), ZEB1 (1:1000, Novus Biologicals, CO), and ß-actin (1:5000, Sigma, MO). Quantification of protein expression relative to total receptor or ß-actin was obtained using Image J software.

#### Multiplex array

Each tumor sample was homogenized directly in lysis buffer for protein extraction. 2ug/ul of sample was used. A multiplex inflammatory multiple cytokines protein array was used (V-PLEX Proinflammatory Panel1 mouse kit, Cat. Number: K15048D) for ELISA analysis.

### Immunofluorescence/Immunohistochemistry

For analysis of frozen sections of mouse tumor tissues, the tumors were excised and frozen in optimal cutting temperature compound (Tissue-Tek). Transverse tumor sections, 10 μm thick, were immunostained with specific antibodies. To obtain mosaic images of tumor sections, an Olympus FV1000 confocal laser-scanning microscope was used. A 10x air objective acquired 1260-μm square tiles, and an automated stage scanned throughout the entire cross-section of tumor tissue. The imaged tiles were stitched into a final mosaic image using Olympus software. Antigen expression was quantified by measuring the area occupied by the stain of interest normalized by the area of DAPI-stained nuclei (i.e., unitless), and analyzed using a custom algorithm in MATLAB (The MathWorks). Identical settings and thresholds for analysis were used for all tumors. Antibodies used for immunofluorescence were the following: Collagen-I [LF-68 antibody, 1:50 dilution, provided by Dr Larry Fisher (NIDCR)]; Hyaluronan (biotinylated hyaluronan proteoglycan fragment, 385911, Calbiochem, 1:200 dilution); αSMA (C6198 antibody, Sigma, 1:500 dilution); and F4/80 (MCA497A488 antibody, ABDserotec, 1:200 dilution). Cy3-, Cy5- or FITC-conjugated secondary antibodies were used for the detection of signals by confocal microscopy. Slides were counterstained with DAPI for nuclear staining.

### MMP activity assay

Each tumor sample was homogenized directly in lysis buffer for protein extraction. A standard commercial assay (Abcam ab112146) was used to detect the general activity of MMPs in the tumor samples. Tumor lysates were incubated with a fluorescent substrate [fluorescence resonance energy transfer (FRET) peptide] for 1, 2, and 16 hours, and fluorescence was measured using a fluorescent microplate reader.

### Flow Cytometry

Tumor-bearing mice were perfused through intracardiac injection of PBS and sacrificed. Pancreatic tumor tissues were harvested, minced, and digested at 37°C for 1 h with DMEM containing collagenase type 1A (1.5 mg/mL), hyaluronidase (1.5 mg/mL), and DNase (2 ug/mL). The digestion mixtures were filtered through 70-μm cell strainers. Single-cell suspensions were incubated with rat anti-mouse CD16/CD32 mAb for blocking and stained with fluorochrome-conjugated antibodies in cold buffer (1% BSA, 0.1% NaN3 in PBS). 7-amino-actinomycin D (7AAD) reagent (eBioscience) was added to the stained tubes per manufacturer’s instruction immediately before running the flow analysis. Flow cytometry data were acquired on an LSRII flow cytometer (Becton Dickinson) and were analyzed with FACSDiva software. FSC-A vs. FSC-W and SSC-A vs. SSC-W were applied to discriminate the doublet/aggregated events. The following monoclonal anti-mouse antibodies were used: CD45-PE, CD45-PE-Cy7, CD45-FITC, CD11b-APC-Cy7, CD11b-APC, F4/80-APC (BD Biosciences) and F4/80-FITC and F4/80-PE (eBioscience).

### Macrophage isolation

After cutting the tumor tissue into pieces 0.5 mm in diameter, tumors were dissociated with collagenase and hyaluronidase for one hour. Dissociated tumors were washed with sorting buffer (0.5% BSA in PBS) and passed through cell strainers before they were blocked using Fcr and subsequently incubated with a F4/80 biotinylated primary antibody (eBiosciences) for 15 min. Anti-biotin conjugated magnetic beads (Miltenyl Biotech) were added to the cell suspension and incubated for 15 min before exposing the cells to magnetic columns (MACS LS-columns from Miltenyl Biotech) for cell separation.

### Statistical analysis

Statistical differences between groups were assessed by a two-tailed Student t-test. Two-way analysis of variance was used when comparing body weight over time between the control and metformin-treated groups. The incidence of metastasis and wall invasion was analyzed using chi-square. All statistical analyzes were performed using Prism Graphpad software. All results were considered statistically significant when the P value was less than 0.05 when calculated with the appropriate statistical test. Results are presented as the mean ± standard error.

## Results

### Metformin reduces desmoplasia in mouse and human PDACs

**Fig 1:**
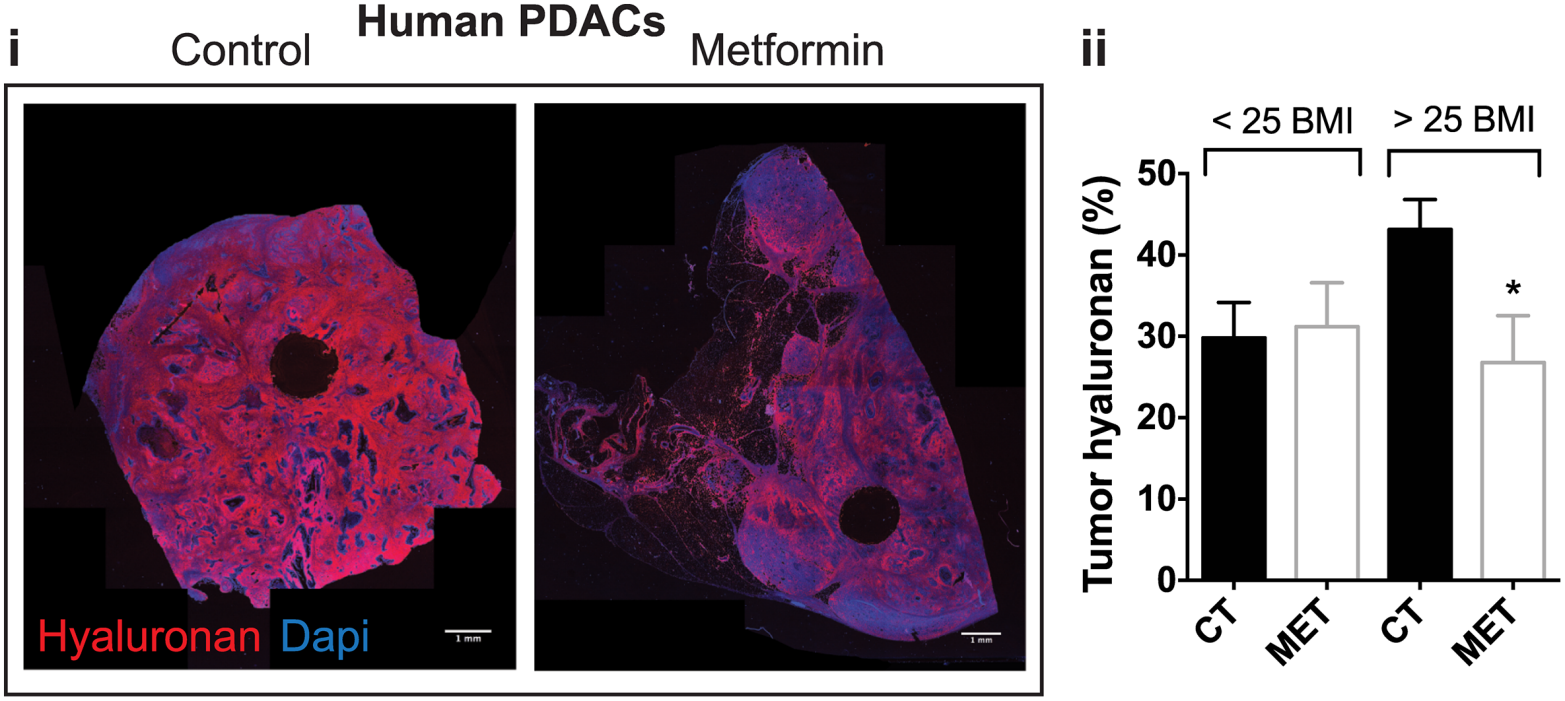
Metformin treatment associates with reduced hyaluronan levels in human pancreatic cancers in overweight/obese patients. (i) Representative histology images showing the effect of metformin on tumor hyaluronan levels in normal weight [Body mass index (BMI)<25)] or overweight/obese patients (BMI>25) (n = 22 controls, 7 metformin). (ii) Immunohistochemical analysis of total tumor hyaluronan levels. Metformin decreases the hyaluronan-positive area fraction (%) in overweight/obese patients. Data are presented as the mean ± standard error. * p < 0.05 vs. control in patients with BMI >25.

We have shown that the reduction of desmoplasia—lowering ECM components such as HA and collagen-I—in PDACs potentiate anti-tumor treatments [41–43]. Hence, we examined the relationship between metformin treatment and desmoplasia in PDACs in chemo/radiotherapy naïve PDAC surgical samples. We found that in overweight or obese patients under treatment with metformin, levels of HA were 30% lower than in patients not taking metformin (Fig 1i and 1ii). Interestingly, no difference in HA levels was observed in metformin treated patients of normal weight. On the other hand, treatment with metformin appeared to have no impact on collagen-I expression in either body weight group (S1 Fig).

**Fig 2:**
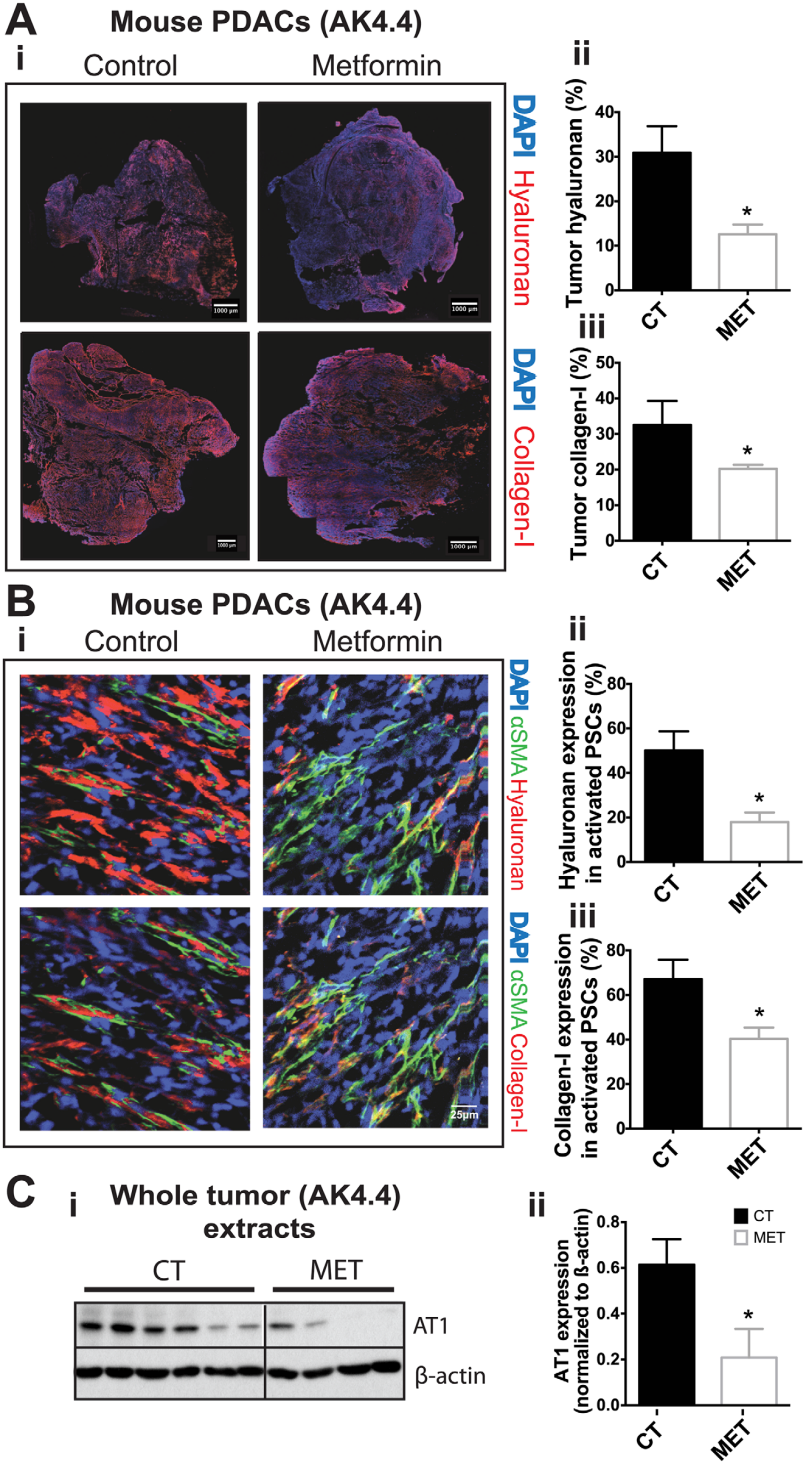
Metformin reduces ECM and AT1 expression in AK4.4 pancreatic cancer model in overweight/obese mice. After 10 weeks of high-fat diet, AK4.4 tumors were orthotopically implanted in obese FVB mice. Animals were randomly assigned to metformin in drinking water (300 mg/kg) or no treatment at day 7 until day 21 when tumors were collected. (A-i) Representative immunohistochemistry images showing the effect of metformin on tumor hyaluronan and collagen-I levels in AK4.4 tumors (n = 3–4). (A-ii) Quantification of hyaluronan expression in AK4.4 tumors. Metformin decreases the hyaluronan-positive area fraction (%). (A-iii) Quantification of collagen-I expression in AK4.4 tumors. Metformin decreases the collagen-I-positive area fraction (%). (B-i) Representative immunohistochemistry images showing the effect of metformin on expression (co-localization) of hyaluronan and collagen-I levels in activated (αSMA-positive) pancreatic stellate cells (PSCs) in AK4.4 tumors (n = 3–4). (B-ii) Quantification of hyaluronan expression in PSCs. Metformin decreases the percentage of activated PSCs expressing hyaluronan. (B-iii) Quantification of collagen-I expression in PSCs. Metformin decreases the percentage of activated PSCs expressing collagen-I. (C-i) Representative Western blots for angiotensin-II receptor-1 (AT1) expression in AK4.4 tumors. ß-actin is used as a control for protein loading. (C-ii) Densitometric analysis of AT1 expression normalized to ß-actin. Metformin decreases the expression of AT1. Data are presented as the mean ± standard error. * p < 0.05 vs. control.

To evaluate if we could recapitulate these findings in pre-clinical models, we used FVB and C57BL/6 mice fed with a high-fat diet. We, along with others have shown that a high-fat diet induces obesity and metabolic abnormalities typical of DM2, elevated glucose, insulin and IGF-1 [50, 57–60] in these strains. After 10 weeks on the high-fat diet, AK4.4 and PAN02 tumors were orthotopically implanted in obese FVB and C57BL/6 mice, respectively. The animals were randomly assigned to metformin in drinking water (300 mg/Kg) or no treatment at day 7 until day 21 when plasma and tumors were collected. Treatment with metformin correlated with reduced expression of HA by 64% (Fig 2Ai and 2Aii) and of collagen-I by 35% (Fig 2Ai and 2Aiii) in AK4.4. tumors. Furthermore, the percentage of activated PSCs (as determined by the expression of alpha-smooth muscle antigen, αSMA) that co-express HA and collagen-I decreased by 58 and 38%, respectively (Fig 2Bi–2Biii). In a second, less desmoplastic model (PAN02), metformin decreased the expression of HA by 40% and of collagen-I by 22%, although it did not reach statistical significance (S2i–S2iii Fig). Nonetheless, metformin significantly reduced the density of collagen-I positive activated PSCs in tumors by 54% in this model (S2v Fig). The density of HA positive activated PSCs in PAN02 tumors was also reduced by 57% (S2iv Fig) but did not reach significance. We have shown previously that Angiotensin-II receptor 1 (AT1) is critical for HA and collagen-I production in PDACs [43]. Indeed, we observed that metformin was able to reduce the expression of AT1 (Fig 2C). Taken together, these data indicate that metformin reduces desmoplasia in PDACs in overweight/obese hosts.

### Metformin affects desmoplasia by directly reducing TGF-β signaling and production of collagen-I/HA by PSCs

**Fig 3:**
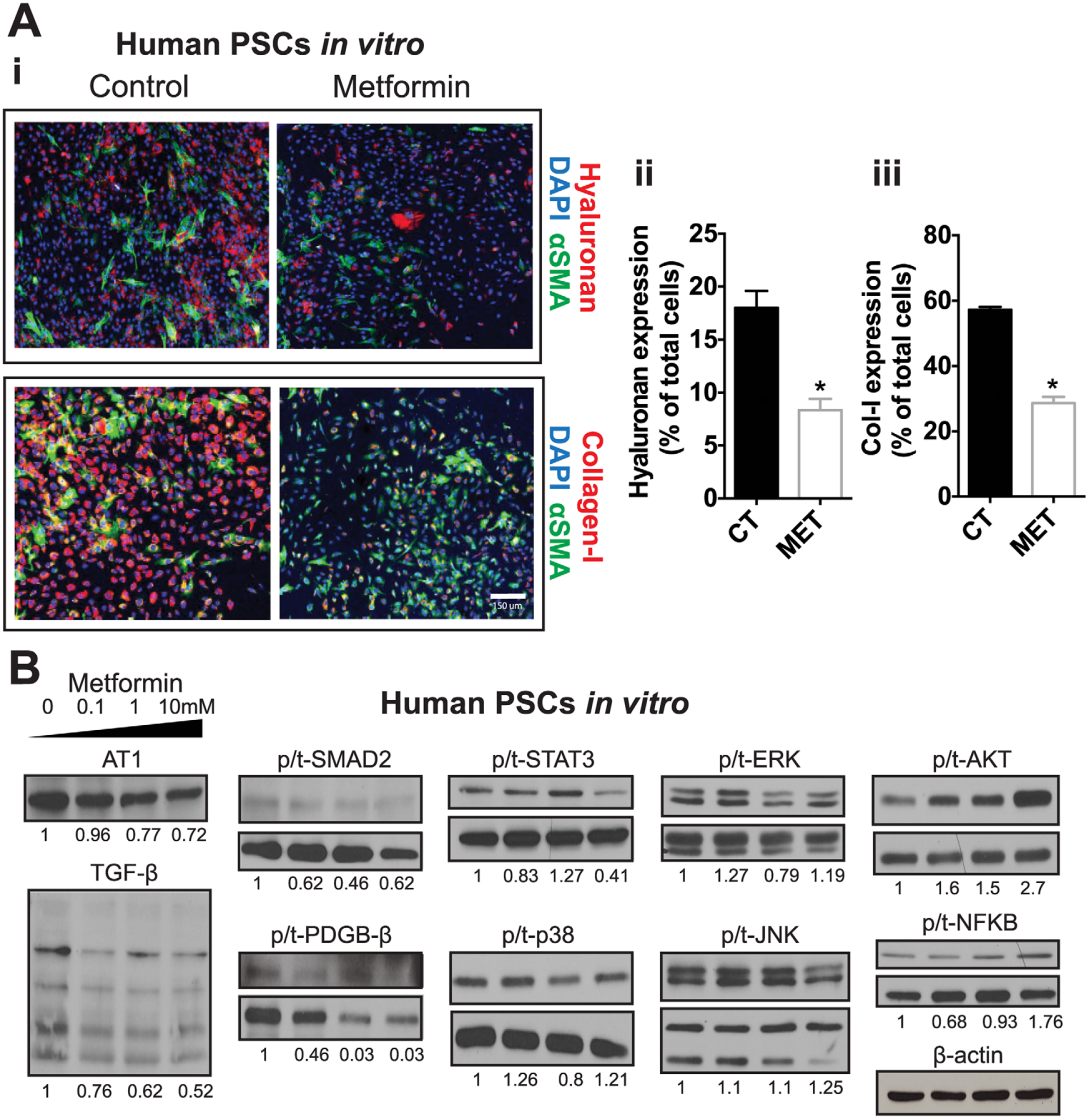
Metformin reduces collagen-I/hyaluronan production by pancreatic stellate cells. (A) PSCs were incubated *in vitro* with metformin (1 mM) for 48h. (A-i) Representative immunocytochemistry images showing the effect of metformin on tumor hyaluronan and collagen-I levels in human pancreatic stellate cells (PSCs) *in vitro* (n = 2). (A-ii) Quantification of hyaluronan expression in PSCs. Metformin decreases the expression of hyaluronan in PSCs. (A-iii) Quantification of the expression of collagen-I in PSCs. Metformin decreases the expression of collagen-I in PSCs. αSMA denotes activated PSCs. (B) Representative Western blots for the expression of fibrosis-related markers and signaling proteins in PSCs treated with metformin at 0, 0.1, 1 and 10mM. Metformin decreases the expression of fibrosis-related markers and signaling proteins in PSCs. Densitometric analysis of protein expression normalized to ß-actin or total protein (in the case of phosphorylated proteins) is depicted as numbers below the representative bands. Data in A are presented as the mean ± standard error. *p < 0.05 vs. control.

Collagen-I and HA are essentially produced by activated PSCs in PDACs [43, 61]. To determine if metformin treatment can directly reduce collagen-I/HA expression in PSCs, we incubated PSCs *in vitro* with metformin. We found that, at a dose (1 mM) that does not substantially affect the viability of PSCs (S3 Fig), metformin decreased the expression of HA and collagen-I (Fig 3Ai–3Aiii). This indicates metformin acts on PSCs to reduce the production of these critical ECM components. Furthermore, we found that metformin (0.1-1mM) reduced expression of AT1, TGF-β and downstream signaling via SMAD-2, as well as PDGF-β, all key players in ECM production by PSCs (Fig 3B). In addition, metformin also affected the activation of canonical signaling pathways that promote PSC activation and fibrosis [62], in particular ERK, p38, and STAT3, although at relatively higher doses (1–10 mM) (Fig 3B). Importantly, in whole tumors, metformin decreased activation of STAT3 in both models, with a trend for reduced p38 activation in PAN02 (S4i–S4iii Fig), suggesting that metformin can accumulate at concentrations high enough to affect these signaling pathways *in vivo*. Taken together, these data indicate that metformin affects desmoplasia by directly reducing AT1/TGF-β/STAT3 signaling and production of collagen-I/HA by PSCs.

### Metformin also improves desmoplasia by preventing recruitment and M2 polarization of macrophages in PDACs

**Fig 4:**
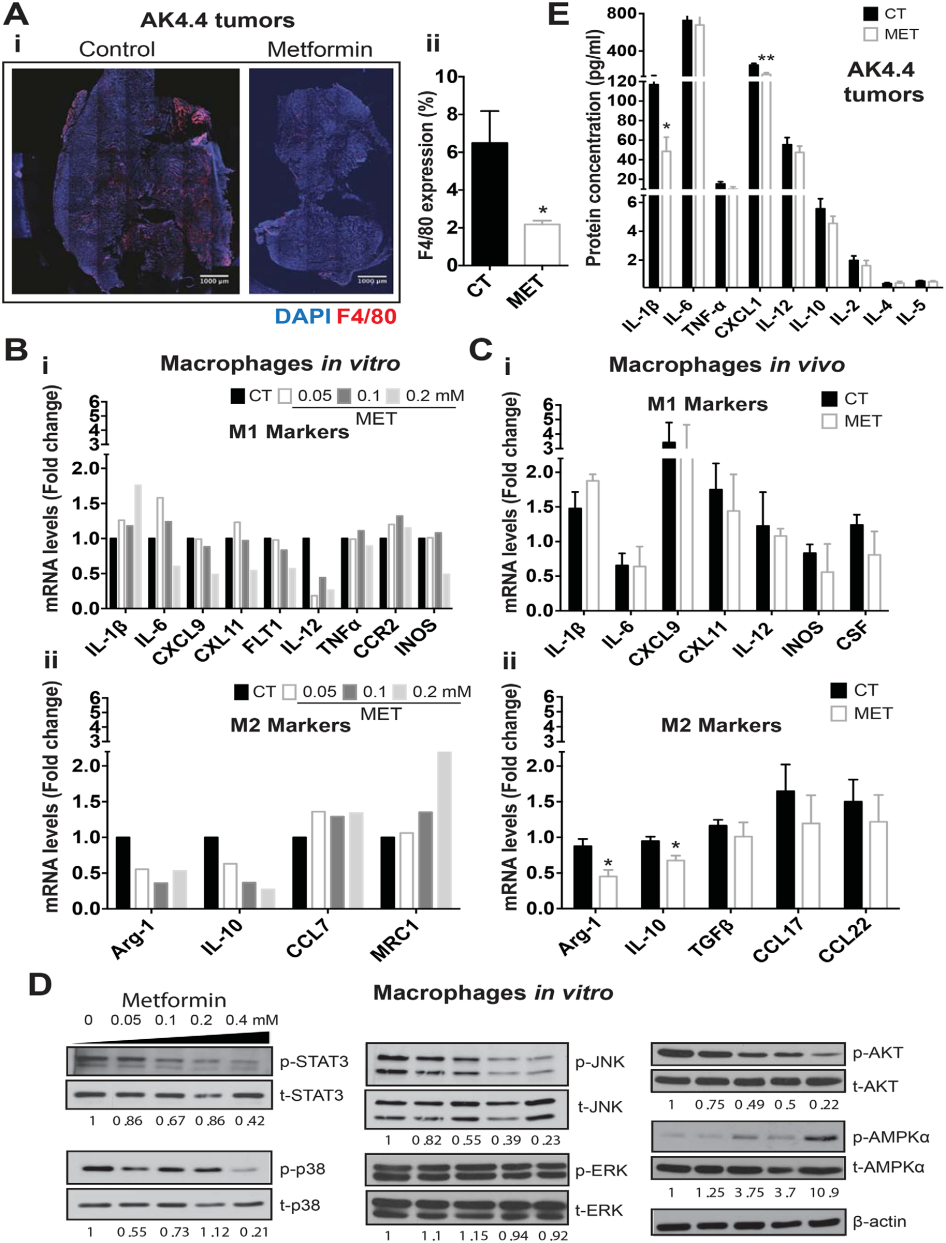
Metformin reprograms TAMs and reduces inflammation in tumors. (A-i) Representative immunocytochemistry images showing the effect of metformin metformin on the expression of F4/80 (immunofluorescence) in AK4.4 tumors (percentage of viable tumor area) (n = 4). (A-ii) Metformin-treated tumors (300 mg/kg in drinking water) had significantly reduced levels of F4/80-positive tumor-associated macrophages (TAMs). (B) Effect of metformin (0–0.2mM) on the gene expression (qPCR) of M1 (i) and M2 (ii) markers in RAW 264.7 cells (mouse leukaemic monocyte-macrophages) *in vitro*. Clinically relevant doses (0.05 mM) of metformin treatment reduces expression of M2 markers in macrophages *in vitro*, including Arg-1 and IL-10. (C) Effect of metformin on the gene expression (qPCR) of M1 (i) and M2 (ii) markers in TAMs isolated from PAN02 tumors *in vivo* (n = 3). Metformin treatment reduced expression of the M2 markers Arg-1 and IL-10 in TAMs *in vivo*. (D) Representative Western blots for the expression of signaling proteins in RAW 264.7 cells treated with metformin at 0, 0.05, 0.1, 0.2 and 0.4 mM. Metformin decreases the activation of signaling pathways and increased activation of AMPKα on RAW cells. Densitometric analysis of protein expression normalized to ß-actin or total protein (in the case of phosphorylated protein) is depicted as numbers below the representative bands. (E) Effect of metformin on the protein expression of major cytokines in AK4.4 tumors (n = 4–5) using multiplex protein array. Metformin treatment associated with reduced IL-1ß and CXCL1 expression in tumors. Data are presented as mean ± standard error in A, C and E. * p < 0.05, ** p < 0.01 vs. control.

The inflammation that occurs in PDACs is a major component of desmoplasia [63]. In particular, tumor-associated macrophages (TAMs) are a major source of cytokines that aggravate desmoplasia in PDACs [64] and negatively affect disease outcome [65]. Therefore, we determined here the effect of metformin on TAM infiltration in tumors. We found that TAM levels were 60% lower with metformin treatment in the AK4.4 model (Fig 4A). They also tended to be lower (~30%, not significant) in the PAN02 model (S5A Fig). To determine a direct effect of metformin on macrophages, we incubated macrophages with metformin *in vitro* for 48h at increasing concentrations. We found that metformin affected the viability of macrophages at doses of 0.4 mM or higher (S6 Fig). Metformin at a concentration of 0.05 mM (similar to the concentration measured in plasma of patients taking metformin) reduced M2 markers such as Arg-1 and IL-10, while metformin at doses higher than 0.2 mM reduced both M1 and M2 markers (Fig 4B). In flow-sorted TAMs from PAN02 tumors *in vivo*, we indeed confirmed an effect of metformin on the expression of M2 markers Arg-1 (~1/2) and IL-10 (~2/3) without significantly affecting the expression of M1 markers (Fig 4C). We then sought to understand how metformin affects these cells. Several canonical and non-canonical signaling pathways can be activated in PDACs during inflammation and promote expression of M2 markers on TAMs [66–69]. Consistent with the effects on TAMs, metformin reduced activation of STAT3, JNK, AKT and p38 in macrophages *in vitro* at concentrations lower than 0.2 mM (Fig 4D). As mentioned above, in whole tumors metformin decreased activation of STAT3 in both models, with a trend for reduced activation of p38 in PAN02, in line with our *in vitro* results (S4i–S4iii Fig). It has been shown that STAT3 activity is decreased by metformin via activation of metabolic energy sensor AMP-activated protein kinase (AMPK) in multiple cell types [70]. Indeed, we found that the inhibitory effects of metformin on STAT3 signaling in macrophages *in vitro* associated with activation of AMPKα and downstream enzyme Acetyl-CoA Carboxylase (ACC) (the latter evident only in serum added media) in these cells (Fig 4D and S7 Fig). Taken together, these data indicate that metformin reduces TAM infiltration as well as expression of M2 markers, which may be mediated at least in part via AMPK/STAT3 signaling inhibition in macrophages. In addition, inflammation in tumors is characterized by an excess of inflammatory cytokines that promote desmoplasia, and metformin has been shown to affect multiple inflammatory mediators [34, 35]. Here, we found that metformin reduced the expression of IL-1β and CXCL1 in AK4.4 tumors (Fig 4E). A reduction of IL-1β after metformin treatment also occurred in the PAN02 tumor model (S5Bi and S5Bii Fig). In addition, a broader panel of inflammation-related genes in AK4.4 tumors revealed that metformin reduced multiple genes involved in TAM recruitment and function (S5C Fig). In conclusion, we found that metformin reduces the production of desmoplastic cytokines (e.g., IL-1β) as well as infiltration and M2 polarization of TAMs, which may be mediated at least in part via AMPK/STAT3 signaling inhibition in macrophages.

### Metformin reduces ECM remodeling, EMT, and metastasis

**Fig 5:**
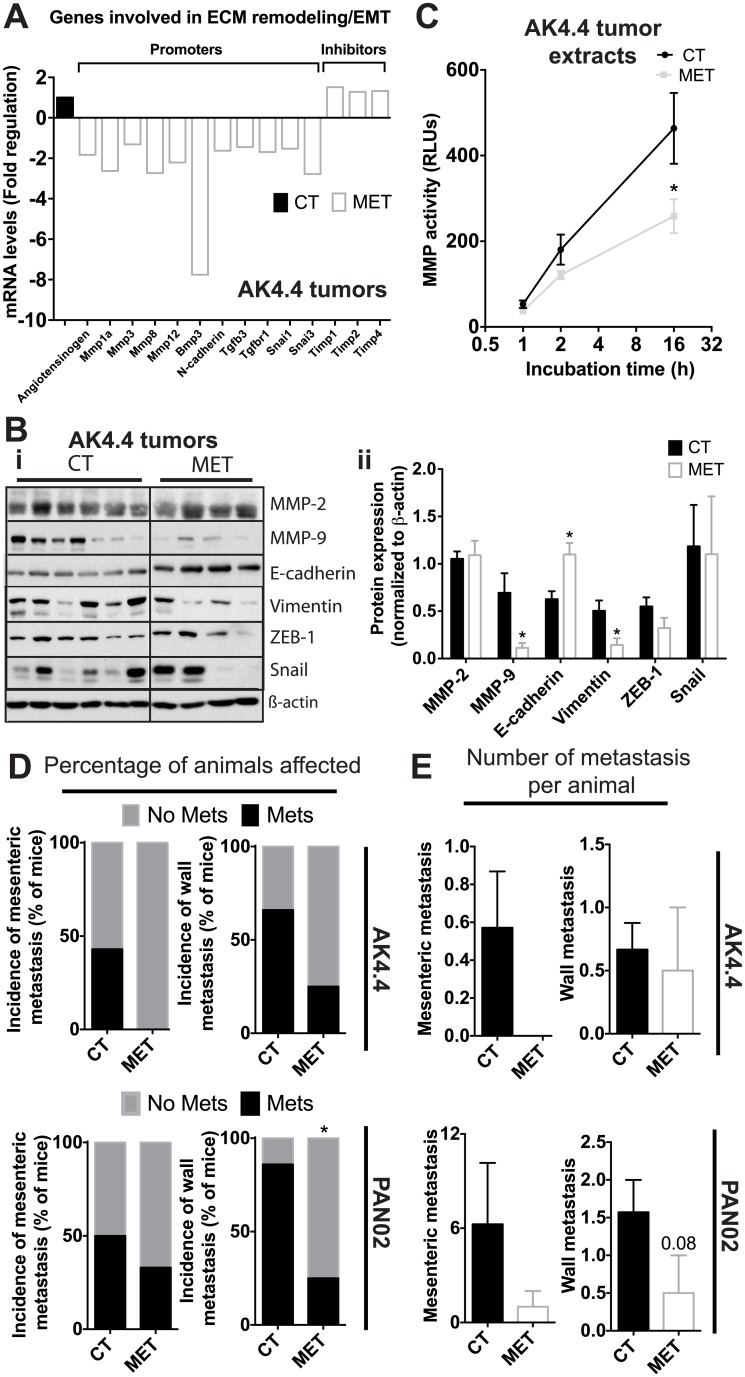
Metformin reduces ECM remodeling, EMT, and metastasis in a PDAC mouse model. (A) Expression of genes associated with extra-cellular matrix (ECM) remodeling, epithelial-to-mesenchymal transition (EMT) and inflammation in AK4.4 tumors from control and metformin-treated mice. Data normalized to control group. 3–4 samples per group pooled in one single PCR array plate. Metformin reduces the expression of pro-tumor genes and increases the expression of anti-tumor genes. (B-i) Representative Western blots showing the effect of metformin (300 mg/Kg) on MMPs and EMT markers in AK4.4 tumors. (B-ii) Densitometric analysis of protein expression normalized to ß-actin. Metformin decreases the expression of MMP-9 and vimentin and increases the expression of e-cadherin in AK4.4 tumors. (C) MMP activity in AK4.4 tumor protein extracts from control and metformin-treated mice (n = 3–4). Metformin decreases the activity of MMPs. (D) Effect of metformin on the percentage of mice affected (incidence) with mesenteric (peritoneal) and abdominal wall (retroperitoneal) metastasis in AK4.4 and PAN02 models (n = 3–8). Metformin reduced the percentage of mice that develop wall metastasis in the more metastatic model (PAN02 model) and induced a tendency for reduced wall as well as mesenteric metastasis in the less metastatic AK4.4 model. (E) Effect of metformin on the number (average) of mesenteric (peritoneal) and abdominal wall (retroperitoneal) metastasis per mouse in the AK4.4 and PAN02 models (n = 3–8). Metformin reduced the number of wall metastasis in the PAN02 model. There were also trends for fewer mesenteric metastasis in AK4.4 and PAN02 tumors. Data in B, C and E are presented as the mean ± standard error. *p < 0.05 vs. control.

In addition to producing ECM components, PSCs also promote ECM remodeling and EMT to facilitate invasion and metastasis [43, 71, 72]. Hence, we determined whether the effects on PSCs by metformin also extend to these processes. Indeed, metformin reduced the expression in AK4.4 tumors of multiple genes involved in ECM remodeling (including MMPs) and EMT (Fig 5A). In addition, metformin treatment unregulated genes that prevent ECM remodeling (Fig 5A). Though to a lesser extent, similar findings were observed in PAN02 tumors (S8A Fig). At the protein level, we also observed a reduction of metalloproteinase 9 (MMP-9, Fig 5Bi and 5Bii) by 70% in the AK4.4 model with metformin treatment. Average MMP-2 levels were also approximately half (not significant) in metformin-treated PAN02 tumors (S8Bi and S8Bii Fig). Consistently, we confirmed *in vitro* that metformin decreased protein levels of MMP9 in PSCs (S8C Fig). Furthermore, MMP activity in tumors was also decreased in metformin-treated animals compared to control mice (Fig 5C). In addition to ECM remodeling, EMT was also affected. At the protein expression level, the EMT marker vimentin was decreased and E-cadherin was increased in AK4.4 with similar trends in the PAN02 model, confirming reduced EMT (Fig 5Bi and 5Bii, S8Bi and S8Bii Fig). Consistent with these effects on the tumor microenvironment, metformin reduced the incidence of mesenteric peritoneal and retroperitoneal wall metastasis metastasis (percentage of mice affected) (Fig 5D) as well as the average number of metastasis per mouse (Fig 5E). These effects were particularly evident in the more metastatic model PAN02, although similar trends were obtained for the less metastatic AK4.4 model.

### Effects of metformin on desmoplasia are independent of global metabolic effects

As expected, metformin reduced systemic levels of glucose and Insulin/IGF-I in the PAN02 model (S9Ai–S9Aiii Fig), with similar trends occurring for Insulin levels in the AK4.4 model (S9B Fig). At the tumor level, metformin induced a trend for reduced activation and phosphorylation of the insulin-like growth factor receptor-1 (IGFR-1) and downstream mediator insulin receptor substrate 1 (IRS-1) (S9Ci and S9Cii Fig). In addition, activation of ACC was increased by metformin, with similar trends for AMPKα and AMPKß (S9Ci and S9Cii Fig). However, metformin did not affect the activation of metabolic pathways in AK4.4 tumors (S9Ci and S9Ciii Fig), where it more dramatically improved the tumor microenvironment. In addition, the expression of the autophagy marker L3CB was unchanged by metformin in both models (S9Ci–S9Ciii Fig). Furthermore, no change in body weight was observed in either model (S10 Fig). These data suggests that the effects of metformin on desmoplasia and metastasis do not correlate with global activation of metabolic pathways or body weight. On the other hand, as shown above, metformin induced specific changes in metabolic pathways in TAMs. Taken together, our findings indicate that a global change in the activity of metabolic pathways in tumors does not correlate with the effects of metformin on tumor desmoplasia.

## Discussion

Metformin improves cancer outcomes in preclinical models of PDAC [25, 26] and diabetic patients with pancreatic cancer [32, 35] though the underlying mechanisms are not well understood. Hence, there is a need to continue to study and elucidate the mechanisms of action of metformin in PDACs. Fibrosis and inflammation are critical components of the desmoplasia that characterize PDACs [73]. We and others have previously shown that reprogramming the central instigator of the desmoplastic microenvironment—PSCs—can be an effective intervention in the treatment of PDACs [43, 61, 74]. Furthermore, metastases in PDACs are facilitated by the active desmoplastic fibro-inflammatory microenvironment that promotes ECM remodeling, EMT and tumor invasion [43–45, 75]. We uncovered in this study a previously unknown role of metformin on the activity of PSCs, TAMs, tumor fibrosis and inflammation, and how it impacts systemic dissemination of the disease. We found that in overweight and obese patients—which appear to have increased levels of ECM components in tumors -, metformin treatment reduced tumor levels of HA. We confirmed that metformin robustly affected HA as well as collagen-I, though to a lesser extent, in preclinical obese/diabetic mouse models of syngeneic PDACs. Furthermore, we found that the alleviation of desmoplasia occurred due to a direct effect on HA and collagen-I production by PSCs, which was associated with a reduction of AT1/PDGF-ß expression and TGF-ß/SMAD-2 signaling. Although the precise mechanisms for the preferential effect of metformin on HA over collagen-I in all preclinical models and human cancer patients studied so far are not clear, it is an intriguing and potentially important finding. Nonetheless, we have recently observed that HA plays an equally important role as collagen-I in reducing therapy delivery and efficacy in PDACs [43, 76].

In addition to reducing fibrosis, we found here that metformin reduces the production of pro-metastatic cytokines. In both tumor models, metformin reduced the secretion of IL-1ß, which has been shown to promote metastasis in a PDAC model [30]. IL-1β in tumors is typically produced by PSCs, inflammatory and tumor cells, is involved in macrophage recruitment and PSC activation, and both IL-1β and CXCL1 worsen desmoplasia [77–80]. Consistently, it has been shown that IL-1β mediates at least in part the effects of metformin on the malignant transformation of mammary epithelial cells [81]. Furthermore, we found that metformin reduced the levels of chemokines involved in TAM recruitment and function (e.g. CSFs, CCL3) [82–84], and consistently, metformin reduced the recruitment of TAMs and their expression of M2 markers *in vivo* and *in vitro* at clinically relevant doses. Our data corroborate the previous findings of Karnevi et al showing that metformin shifts macrophage polarization *in vitro* [17]. STAT3 promotes polarization of TAMs to an M2 phenotype [85], and It has been shown that STAT3 activity is decreased by metformin [81] in part via activation of AMPKα in multiple cell types [70, 86]. We found here that the effects of metformin on macrophage polarization associated with activation of AMPKα/ACC and reduction of STAT3 signaling.

Activated PSCs and M2 TAMs have been shown to promote ECM remodeling and EMT [87, 88]. Here we found that, consistent with the effects on these cells, metformin reduces ECM remodeling and EMT. This is also in line with a recent report describing the modulation of EMT by metformin in the PAN02 model [89], and with the finding that metformin impeded TGF-ß-promoted EMT in breast cancer cells [90]. Importantly, ECM remodeling and EMT have been shown to promote tumor invasion and metastasis [43–45, 75], and as expected we observed a decrease in metastasis in mice treated with metformin.

The modulation of systemic and local metabolism has been the major focus of studies evaluating the effect of metformin in PDAC. Metformin could improve systemic levels of Insulin/IGF-1 and glucose, and affect Insulin/IGF-1 signaling and AMPK/ACC activation. However, we found that this only occurred (and mildly) in one of the models (PAN02). This suggests that the effects of metformin on desmoplasia do not correlate with global activity of metabolic pathways. In fact, despite earlier reports suggesting that IGF-I may be involved in cancer risk and outcome [91], subsequent clinical studies failed to establish anti-IGF-I agents as cancer therapeutics [92]. In addition, there is no convincing evidence for a carcinogenic role of any insulin derivative currently used in therapy for diabetes [93]. Similarly, the beneficial effects of metformin did not correlate with levels of blood sugar in patients [33], and in a pre-clinical model Franco and colleagues have shown that the effects of metformin may be more dependent on the direct effect on tumors rather than on systemic metabolism [94]. In addition, despite the report that autophagy can be affected by metformin to reduce tumor progression [95, 96], we found no evidence of this in our study. Of note, metformin did not affect body weight through the experiment (S10 Fig), suggesting a body weight-independent effect on tumor growth.

Importantly, two very recent studies—one retrospective [97] and the first prospective study [98] indicated that metformin might not be uniformly beneficial. The benefit in some but not all studies suggests that a subset of tumors may not respond to metformin and that a careful selection of patients may be required for metformin to be effective. We found that metformin’s effect on desmoplasia in patients only occurred when their BMI was higher than 25 (overweight and obese patients). This indicates that metformin may not be beneficial in normal weight patients, and suggests that BMI should be explored as a potential biomarker of response to this drug.

## Conclusion

**Fig 6:**
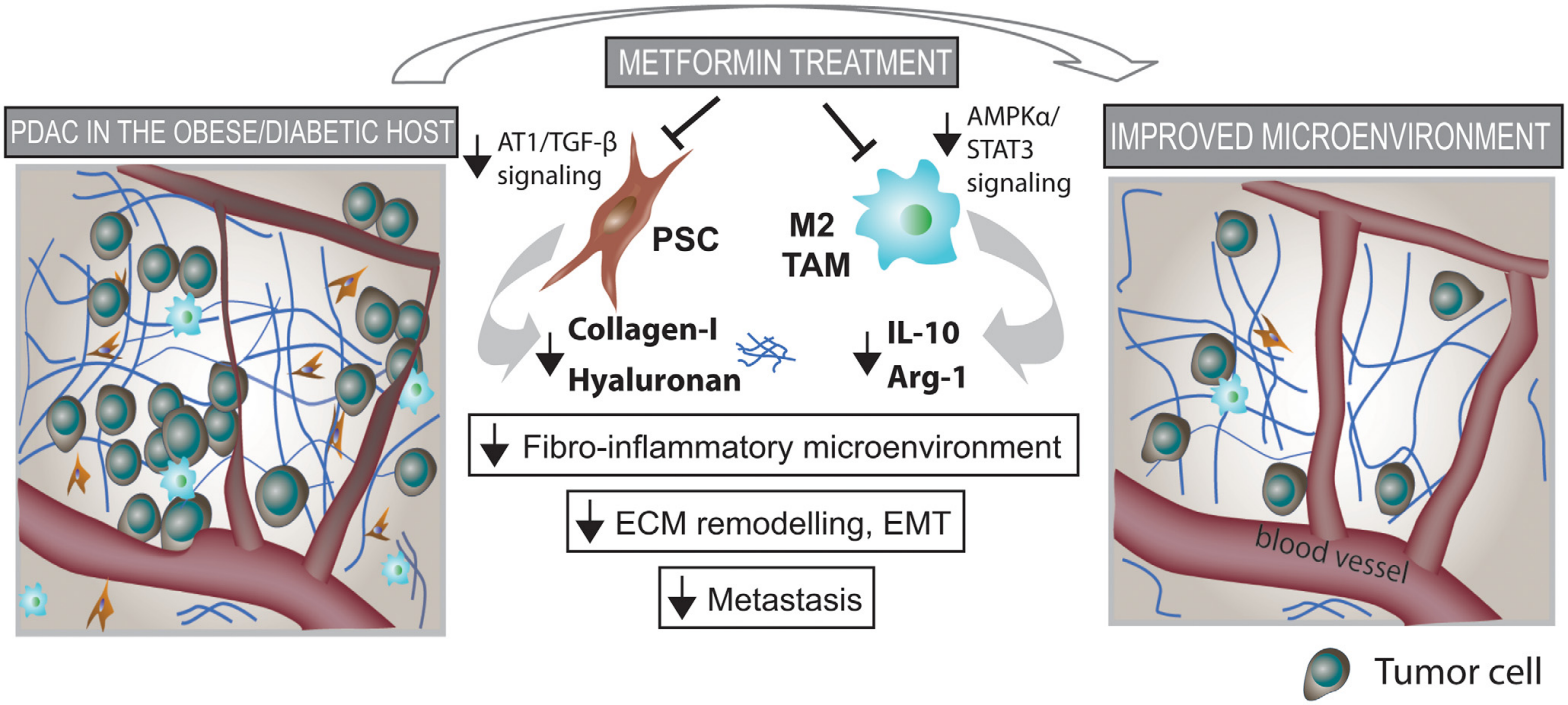
Metformin reprograms pancreatic stellate cells (PSCS) and tumor-associated macrophages (TAMs), alleviates the fibro-inflammatory tumor microenvironment and reduces metastasis. Metformin treatment reduces collagen-I and HA production by PSCs, leading to decreased fibrosis in PDACs. Metformin treatment also reduces cytokine production, infiltration and M2 polarization of TAMs, leading to decreased inflammation. This associated with improved desmoplasia and reduced extracellular matrix (ECM) remodeling, epithelial-to-mesenchymal transition (EMT), and metastasis.

In conclusion, this study indicates that in an overweight/obese condition, metformin reprograms the fibro-inflammatory tumor microenvironment and ultimately reduces metastasis. We found that metformin directly reduces AT1/PDGF-ß and TGF-ß signaling and ECM production by PSCs—preferentially HA. Metformin also reduces inflammation—another key element of desmoplasia—through reduction of cytokine production, and recruitment and M2-polarization of TAMs. This was associated with AMPK activation and STAT3 signaling inhibition in macrophages. Finally, the alleviation of desmoplasia by metformin was associated with reduced ECM remodeling, EMT and systemic metastasis (Fig 6). Importantly, the effects on desmoplasia observed in human samples seem restricted to an overweight/obese population, which appear to have tumors with increased content of ECM components. With nearly 200 trials ongoing to address the effect of metformin on diabetic and non-diabetic cancer patients, understanding the yet elusive mechanisms of action of metformin may provide an opportunity to uncover potential biomarkers of response and define strategies of patient stratification for the judicious use of this highly promising yet generic drug.

## Supporting Information

S1 Fig Metformin treatment does not reduce collagen-I levels in human pancreatic cancers. Effect of metformin on tumor collagen-I levels in normal weight [Body mass index (BMI)<25)] or overweight/obese patients (BMI>25). (i) Immunohistochemical analysis of total tumor collagen-I levels. Metformin treatment does not alter tumor collagen-I-positive area fraction (%) (n = 23 controls, 6 metformin). Data are presented as the mean ± standard error.
(EPS)

S2 Fig Metformin reduces density of ECM producing PSCs in PAN02 tumors. (i) Representative immunohistochemistry images showing the effects of metformin on α-SMA, hyaluronan and collagen-I levels in PAN02 tumors. (ii) Immunohistochemical analysis of total α-SMA/hyaluronan double positive cells. Metformin induced a tendency for decreased area fraction (%) of hyaluronan-positive activated PSCs (n = 5–6). (iii) Immunohistochemical analysis of total α-SMA/collagen-I double positive cells. Metformin decreases the area fraction (%) of collagen-I-positive activated PSCs (n = 5–6). Data are presented as the mean ± standard error. *p < 0.05 vs. control.
(EPS)

S3 Fig High doses of metformin reduce the viability of PSCs *in vitro*. Effect of metformin on human PSC viability. PSCs were incubated with metformin at increasing doses for 48 h, and MTT viability assay was performed. At high doses (>7.5mM), metformin significantly reduces cell viability. Values are the mean ± standard error. *p < 0.05, **p < 0.01, ***p < 0.001 vs. untreated control.
(EPS)

S4 Fig Metformin reduces pSTAT3 expression in PDACs. (i) Representative blots showing the effect of metformin on phospho and total p38, STAT3, JNK and NFKB expression in AK4.4 and PAN02 tumors. ß-actin is used as a control for protein loading. (ii) Densitometric analysis of protein expression normalized to total levels of the protein of interest. Metformin decreases the expression of p-STAT3 in both tumor models. Data are presented as the mean ± standard error. * p < 0.05 vs. control.
(EPS)

S5 Fig Metformin reduces inflammation in a second PDAC mouse model (PAN02) and additional gene expression data on AK4.4 model. (A) Effect of metformin on the levels of CD45(+)CD11b(+)F4/80(+) TAMs in PAN02 tumors (percentage of total viable cells as assessed by flow cytometry) (n = 4–7). We observed a tendency for reduced numbers of TAMs with metformin. (B) Effect of metformin on the protein expression of major cytokines in the PAN02 tumor model. Metformin treatment associates with reduced IL-1ß expression in tumors (i) and plasma (ii) (n = 6–7). (C) Expression of genes associated with inflammation in AK4.4 tumors from control and metformin-treated mice. Data normalized to control group. 3–4 samples per group pooled in one single PCR array plate. Metformin reduces the expression of pro-tumor genes and increases the expression of anti-tumor genes. Data are presented in A and B as the mean ± standard error. *p < 0.05 vs. control.
(EPS)

S6 Fig Metformin reduces the viability of macrophages *in vitro*. Effect of metformin on macrophage viability *in vitro*. Macrophages were incubated with metformin at increasing doses for 48 h, and MTT viability assay was performed. At doses > 0.2mM metformin significantly reduces cell viability. Data are presented as the mean ± standard error. * p < 0.05, ** p < 0.01 vs. untreated control.
(EPS)

S7 Fig Additional metabolic effects of metformin on macrophages *in vitro*. Representative Western blots for the expression of additional metabolic markers (AMPK-ß and ACC) on RAW 264.7 (mouse leukaemic monocyte-macrophage) cells treated with metformin at 0, 0.05, 0.1, 0.2 and 0.4 mM. Metformin increased the activation of ACC when serum was present in the media. Densitometric analysis of protein expression normalized to total protein is depicted as numbers below the representative bands.
(EPS)

S8 Fig Effects of metformin on ECM remodeling and EMT in a second PDAC mouse model (PAN02) and *in vitro*. (A) Expression of genes associated with ECM remodeling, EMT and inflammation in PAN02 tumors from control and metformin-treated mice. Data normalized to control group. 3–4 samples per group pooled in one single PCR array plate. Metformin reduces the expression of MMPs and increases the expression of Timp-4. (B-i) Representative blots showing the effect of metformin on MMPs and epithelial-to-mesenchymal transition (EMT) markers in PAN02 tumors. ß-actin is used as a control for protein loading. (B-ii) Densitometric analysis of protein expression normalized to ß-actin. Metformin induced a tendency for decreased MMP-2 and vimentin in PAN02 tumors. (C) Representative Western blot for the expression of MMP-9 on PSCs treated with metformin at 0, 0.1, 1 and 10mM (*in vitro*). Metformin decreased expression of MMP-9. Densitometric analysis of protein expression normalized to total protein is depicted as numbers below the representative bands. Data in B are presented as the mean ± standard error.
(EPS)

S9 Fig The metabolic effects of metformin are not present in both PDAC mouse models. (Ai-iii) Effects of metformin on systemic levels of glucose (i), insulin (ii) and insulin-like growth factor-I (IGF-I) in PAN02 tumor bearing mice (iii) (n = 7–8). Metformin reduces levels of glucose, insulin and induced a trend for reduced levels of IGF-I in circulation. (B) Effects of metformin on systemic levels of insulin in AK4.4 tumor bearing mice (iii) (n = 8) Metformin induced a trend for reduced levels of Insulin in circulation. (C-i) Representative blots showing the effect of metformin on metabolic markers in PAN02 and AK4.4 tumors. ß-actin is used as a control for protein loading. (C-ii) Densitometric analysis of protein expression normalized to total protein or ß-actin in the case of LC3B. Metformin decreases the expression of p-IRS-1 and increased the expression of p-AMPK-β and p-ACC in PAN02 but not in Ak4.4 tumors. Data are presented as the mean ± standard error. *p < 0.05, **p < 0.01 vs control.
(EPS)

S10 Fig Metformin does not affect body weight in tumor-bearing animals. Effect of metformin on body weight after tumor implantation (n = 4–10). Metformin does not affect body weight in tumor-bearing mice. Data are presented as the mean ± standard error.
(EPS)
